# Proton Pump Inhibitors Increase the Risk of Autoimmune Diseases: A Nationwide Cohort Study

**DOI:** 10.3389/fimmu.2021.736036

**Published:** 2021-09-30

**Authors:** Sheng-Hong Lin, Yu-Sheng Chang, Tzu-Min Lin, Li-Fang Hu, Tsung-Yun Hou, Hui-Ching Hsu, Yu-Chuan Shen, Pei-I Kuo, Wei-Sheng Chen, Yi-Chun Lin, Jin-Hua Chen, Chi-Ching Chang

**Affiliations:** ^1^ Division of Allergy, Immunology and Rheumatology, Department of Internal Medicine, Shuang Ho Hospital, Taipei Medical University, New Taipei City, Taiwan; ^2^ Division of Allergy, Immunology and Rheumatology, Department of Internal Medicine, School of Medicine, College of Medicine, Taipei Medical University, Taipei, Taiwan; ^3^ Division of Rheumatology, Immunology and Allergy, Department of Internal Medicine, Taipei Medical University Hospital, Taipei, Taiwan; ^4^ Division of Allergy, Immunology and Rheumatology, Department of Internal Medicine, Wang Fang Hospital, Taipei Medical University, Taipei, Taiwan; ^5^ Division of Allergy, Immunology and Rheumatology, Department of Internal Medicine, Cardinal Tien Hospital, Yonghe Branch, New Taipei City, Taiwan; ^6^ Division of Allergy, Immunology, and Rheumatology, Department of Internal Medicine, Taipei Veterans General Hospital, National Yang-Ming University, Taipei, Taiwan; ^7^ Biostatistics Center, College of Management, Taipei Medical University, Taipei, Taiwan; ^8^ Graduate Institute of Data Science, College of Management, Taipei Medical University, Taipei, Taiwan

**Keywords:** autoimmune disease, proton pump inhibitor, risk, cohort study, epidemiology

## Abstract

**Background:**

Previous study revealed proton pump inhibitors (PPIs) have an effect on gut microbiota. Alteration of the microbiome causes changes of the host immune system and then induces the development of autoimmune diseases (ADs). This study aimed to explore the possible association between PPIs use and ADs.

**Methods:**

This study was conducted using data from the Taiwan National Health Insurance Research Database in the period between 2002 and 2015. We performed multivariate and stratified analysis through the Kaplan-Meier method and Cox proportional hazard models to estimate the association between proton pump inhibitor use and the risk of autoimmune diseases.

**Results:**

Of the 297,099 patients treated with PPI identified, the overall mean (SD) age was 49.17 (15.63) years and 56.28% of the subjects was male. As compared with the non-PPI group, the adjusted hazard ratio (aHR) were higher for incident organ specific ADs such as Graves disease (aHR=3.28), Hashmoto thyroiditis (aHR=3.61), autoimmune hemolytic anemia (aHR=8.88), immune thrombocytopenic purpura (aHR=5.05) Henoch-Schonlein pupura (aHR=4.83) and Myasthenia gravis (aHR=8.73). Furthermore, the adjusted hazard ratio (aHR) were also higher for incident systemic ADs such as ankylosing spondylitis (aHR=3.67), rheumatoid arthritis (aHR=3.96), primary Sjogren syndrome (aHR=7.81), systemic lupus erythemtoasus (aHR=7.03). systemic vasculitis (aHR=5.10), psoriasis (aHR=2.57), systemic scleroderma (aHR=15.85) and inflammatory myopathy (aHR=37.40). Furthermore, we observed no dose-dependent effect between PPI use and the risk of ADs.

**Conclusions:**

Our retrospective population-based cohort study showed that the prescription of proton pump inhibitors is associated with a higher risk of ADs.

## Introduction

Proton pump inhibitors (PPIs), for gastric acid related disorders including gastroesophageal reflux disease and peptic ulcers, are one of the most prescribed medications around the world. These drugs also prevent peptic ulcer diseases in critical patients (1). They also play a fundamental role in the standard regimens used for *Helicobacter pylori* eradication. In other clinical settings, coprescription of PPIs with nonsteroidal anti-inflammatory drugs is used for the prevention to nonsteroidal anti-inflammatory drugs–related gastrointestinal injury, as is accepted by most clinicians. However, much recent research has increasingly identified adverse reactions in patients with long-term prescription of PPIs, including the risks of enteric infection with Clostridium difficile (2), bone fracture (3, 4), osteoporosis (5), malignancy (6), ischemic stroke, myocardial infarction (7), and dementia (8).

Autoimmune diseases (ADs) comprise disorders caused by an imbalance of the immune system, which leads to damage to individual tissues. ADs consist of systemic conditions, such as systemic lupus erythematosus, rheumatoid arthritis, and Sjögren syndrome, as well as single-organ conditions, such as autoimmune thyroid diseases and autoimmune hepatitis. The mechanism underlying the development of ADs remains unknown. Multiple factors (e.g., genetic and environmental factors) affect the risk of ADs. The administration of medications is one of the most important factors in the induction of ADs.

Some medications change the composition of the gastrointestinal microbiota, which may influence human health and cause the development of many diseases (9). The microbiota refers to the millions of microorganisms colonized within the human body that contributes to health and pathology. The first report on the association between PPIs and dysbiosis or disruption of the microbial balance was published in 2008 (10). A meta-analysis reported a statistical relationship between PPIs and bacterial overgrowth in the small intestine (11). Bacterial overgrowth in the small intestine is closely related to dysbiosis (12). Moreover, the dysbiotic disruption caused by administration of PPIs is permanent (13). The dysbiosis was found most often at 4 to 8 weeks after PPIs treatment in patients (14).

The host–microbiota interaction plays an essential role in the host immune system (15). The microbiota influence the immune function. Alteration of the gut microbiome can play a pathogenic role in the development of ADs (16). To investigate the relationship between PPIs use and ADs, we hypothesized that PPIs alter host immune system function and increase the risk of ADs. We conducted a retrospective cohort study to clarify the association between PPIs use and development of ADs.

## Methods

### Data Source

Our data were obtained from the Taiwan National Health Insurance Research Database (NHIRD). In brief, the single-payer mandatory Taiwan National Health Insurance (NHI) program currently covers >99% of the 23 million residents in Taiwan (17). The database contains all individual medical claims data since 1995. Claims data of patients include all ambulatory visits, hospital admissions, treatments, and medications prescribed under the NHI system. Diagnoses were coded according to the International Classification of Diseases (ICD). The accuracy of the diagnoses in the NHIRD, such as diabetes mellitus (18) and malignancy (19), has been validated. The present research was approved by the Institutional Review Board of Taipei Medical University (TMU JIRB-N201908055). Consent waivers were obtained, and all identifying information of patients is anonymized. This c cohort study followed the Strengthening the Reporting of Observational Studies in Epidemiology (STROBE) reporting guideline.

### Study Design and Subjects

This cohort study was retrospectively conducted using the NHIRD. We identified all individuals in the database from January 1, 2002, to December 31, 2015. The entry date was the day when the patients were included to our study, such as the date of the first PPI prescription in PPI users. We identified patients without prescriptions of PPIs during the entire study period as the control group. Patients were followed up from the entry date to the development of AD, death, or the end of the study.

The International Classification of Diseases, Ninth Revision, Clinical Modification (ICD-9-CM) codes were used for the definition of basic characteristics and events of ADs. The Anatomical Therapeutic Chemical (ATC) Classification codes were used for the definition of PPIs, anti-bacterial, antiviral, antifungal, and antituberculosis drugs.

### Exclusion Criteria

We excluded patients who (1) had unknown general data or unknown follow-up time; (2) were less than 20 years old; (3) were diagnosed with ADs before the cohort entry date; (4) had received previous antibiotic, antifungal, anti-tuberculosis, or antiviral agents; and/or (5) had a history of *H. pylori* infection. The *H. pylori* infections were identified as patients receiving therapeutic regimens for *H. pylori* eradication. The *H. pylori* eradication with triple or quadruple therapy was defined as a PPI or H2 receptor blocker, plus clarithromycin or metronidazole, plus amoxicillin or tetracycline, with or without bismuth; details of all eligible *H. pylori* eradication regimens were reported previously (20).

### PPI Exposure

In Taiwan, when applying for reimbursement under the NHI program for PPIs, patients are required to have a diagnosis of reflux esophagitis or peptic ulcer disease through upper gastrointestinal endoscopy or a barium study. In this study, the duration of PPI use was determined according to prescription information contained in NHI claims data. We recorded the drug name, dosage, and start and withdrawal dates of each prescription claim. The PPIs included omeprazole, pantoprazole, lansoprazole, rabeprazole, and esomeprazole (ACT code A02BC), all of which are covered by the NHI program in Taiwan. Dosage of PPIs was presented as the defined daily dose (DDD), which has been established by the World Health Organization as the average maintenance dose per day for a drug used for its main indication in adults. Exposures of PPIs (cumulative dose during follow-up time) were analyzed by treatment continuation determined by the redeemed prescriptions, and the estimated doses were grouped into different cumulative defined daily doses (cDDDs) to assess dose–response effects on hazard ratios and the effects of long-term use on the risks of ADs. The cDDDs was estimated during the study period based on redeeming prescriptions. Furthermore, we divided the patients into four subgroups stratified by cDDDs, as follows: 1–21 DDDs, 22–42 DDDs, 43–98 DDDs, and ≥99 DDDs.

### Comorbidities and Concomitant Medications

We determined potential confounders, associating a given covariate with PPI use on basis of the literature and direct or indirect association with other conditions, such as comorbidities and concomitant medications. According to the literature, these comorbidities and concomitant medications may induce changes in gut microbiota, such as liver cirrhosis (21). We identified comorbidities on the basis of at least two diagnoses of a given disease within 180 days before and after the entry date of our study according to the ICD-9-CM diagnosis codes for liver cirrhosis (ICD-9-CM codes 571, 571.2, 571.5, and 571.6), diabetes (ICD-9-CM codes 250.1–250.9), end-stage renal disease (ICD-9-CM code 585), and malignancy (ICD-9-CM codes 140–208).

### Autoimmune Disease Risk Analysis

The outcomes of systemic and organ-specific ADs were analyzed (see **Supplementary Table 1**). We analyzed the occurrence of ADs in PPI users and nonusers, which was defined as ambulatory or admission to a hospital for ADs after the entry date. Patients with ADs including systemic and organ-specific diseases were identified based on three or more ambulatory care claims or an inpatient setting.

### Statistical Analysis

Patients’ baseline characteristics, including age, sex, coexisting medical conditions, and PPIs doses, were collected. We categorized age in 10-year intervals. Baseline characteristics were compared between PPIs users and nonusers using the chi-square test for categorical variables and the *t*-test for continuous variables, in addition, the Wilcoxon rank-sum test was used for median values of distributions. Baseline was set as the entry date. To understand the risk of autoimmune disorders between PPI and non-PPI users, we calculated the IR (incidence rate) and IRR(incidence rate ratio) from formula, and estimated adj. HR(adjusted hazard ratio) and the 95% confidence interval from Cox regression models to evaluate the occurrence of the all/systemic/organ-specific AD event between PPI and non-PPI users. The baseline information was used for exposure in model adjustment. For model adjustment, we adjusted for sex, age, cancer, diabetes, end-stage kidney disease, and liver cirrhosis. Cumulative incidence rates of ADs were estimated using the Kaplan-Meier method and compared by the log-rank test.

All statistical analyses were performed using SAS for Windows version 9.4 software (SAS Institute, Cary, NC), and a two-sided P value <0.05 was considered statistically significant.

## Results

### Baseline Characteristics

**Figure 1:**
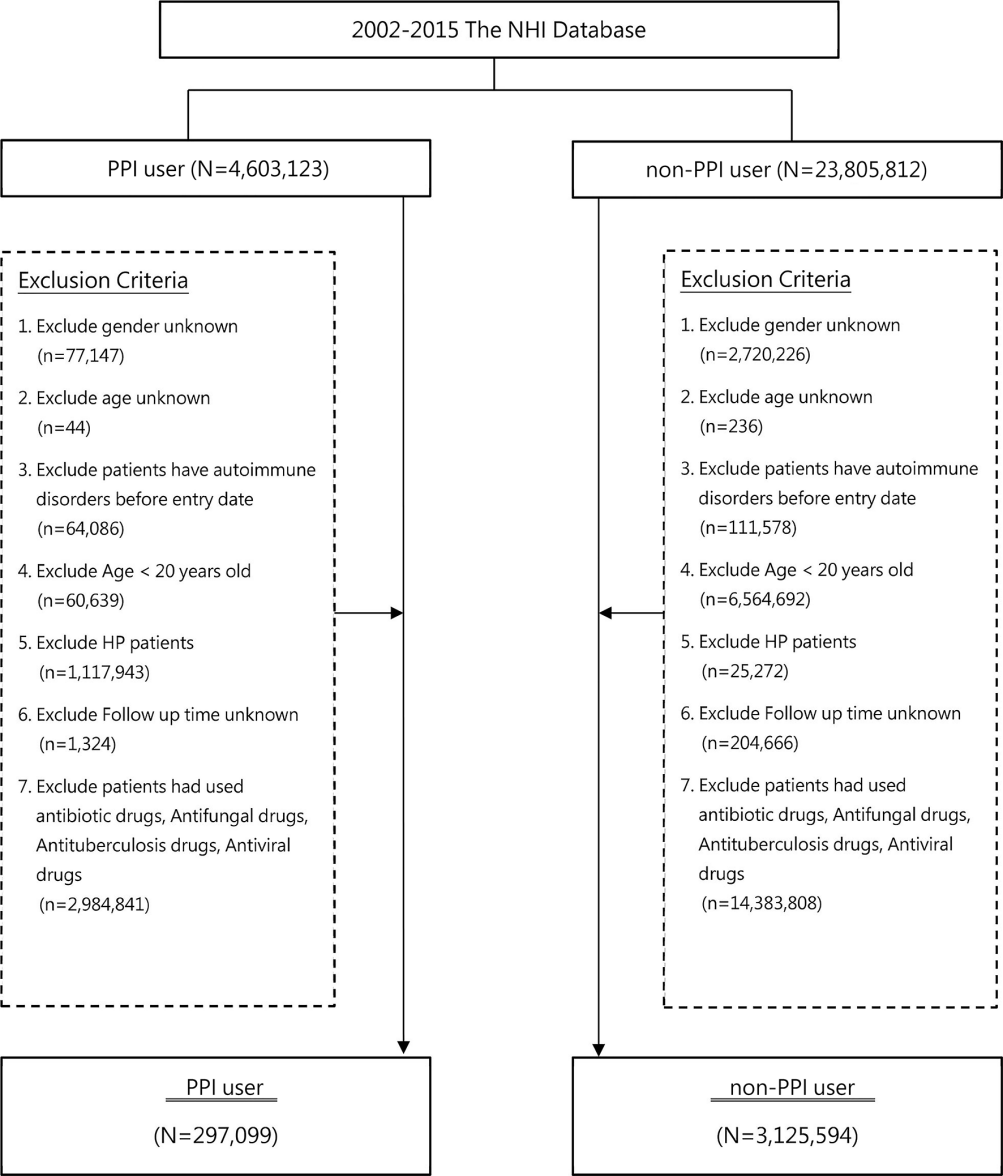
Study flow chart. NHI, nation health insurance; PPI, proton pump inhibitor.

**Figure 1** depicts the study flowchart. A total of 28,408,935 patients were initially identified in this study from the Taiwan NHIRD during 2002–2015. After excluding patients according to the established exclusion criteria, a total of 3,422,693 were identified, including 297,099 in the PPI group and 3,125,594 in the control group. **Table 1** presents baseline characteristics of the PPI group and the control group. The mean ages of patients were 49.17 years (SD, 15.63) and 45.38 years (SD, 16.02) in the PPI and control group, respectively. The event rates of AD development were 0.98% and 0.24% in the PPI and control groups, respectively. Most AD events were systemic diseases with a percentage of 63.49% in PPI users who developed ADs.

**Table 1:** Baseline characteristic of PPI and non-PPI users.

	PPI (n = 297,099)		non-PPI (n = 3,125,594)		P-Value
	n	%	n	%	
**Gender**					0.0043
Female	129,900	43.72%	1,375,250	43.99%	
Male	167,199	56.28%	1,750,692	56.01%	
**Age**					<.0001
20–30	37,209	12.52%	623,753	19.95%	
31–40	57,072	19.21%	690,853	22.10%	
41–50	68,021	22.90%	720,217	23.04%	
51–60	66,238	22.29%	568,056	18.17%	
61–70	38,407	12.93%	276,260	8.84%	
71–80	20,248	6.82%	149,150	4.77%	
≥81	9,904	3.33%	97,653	3.12%	
Mean (SD)	49.17 (15.63)		45.38 (16.02)		<.0001
Median (IQR)	49 (23)		44 (22)		<.0001
**Comorbidities**					
Cancer	13,150	4.43%	50,087	1.60%	<.0001
Diabetes Mellitus	28,708	9.66%	128,694	4.12%	<.0001
End-Stage Renal Disease	4,507	1.52%	11,550	0.37%	<.0001
Cirrhosis of Liver	5,677	1.91%	4,397	0.14%	<.0001
Autoimmune Disorders					
Overall	2926	0.98%	7592	0.24%	<.0001
Systemic	1858	0.63%	4635	0.15%	<.0001
Organ-specific	1075	0.36%	2973	0.1%	<.0001

PPI, proton pump inhibitors.

### Incidence Rates, Ratio, and Adjusted HRs of ADs in PPI and Non-PPI Users

**Figure 2:**
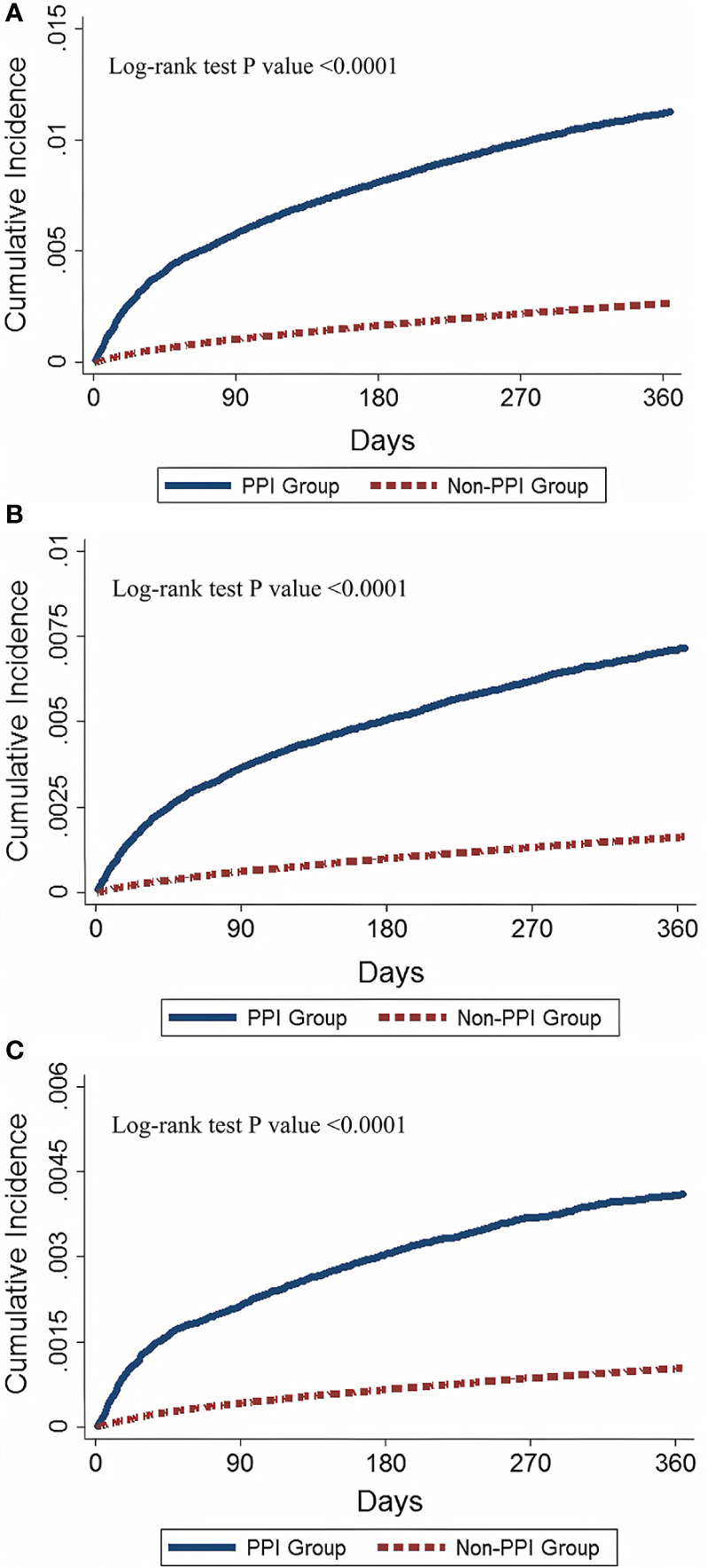
Cumulative incidence of autoimmune diseases in PPI and non-PPI users. **(A)** Cumulative incidence of overall ADs for PPI and non-PPI users **(B)** Cumulative incidence of systemic ADs for PPI users and non-PPI users **(C)** Cumulative incidence of ADs in PPI users and non-PPI users. AD, autoimmune diseases. PPI, proton pump inhibitor.

In the baseline analyses, there were 2926 events in the PPI users and 7592 events in nonusers during the follow-up period of 12 months. The incident rates of ADs were 1219.94 and 274.67 per 100,000 person-years in the PPI users and nonusers, respectively (see **Table 2**). Compared with nonusers, PPI users had a 344% significantly higher absolute risk of AD within a 12-month period (absolute risk ratio, 4.44). After adjusting for age, sex, and comorbidities, we found a higher risk of ADs in PPI users, with an aHR of 3.64 (95% CI, 3.48–3.80; *P* <.0001), compared with nonusers. We also identified a higher risk of ADs in PPI users than in nonusers, restricted the analysis to systemic and single-organ ADs, with aHRs of 4.33 (95% CI, 4.10–4.58; *P* <.0001) and 2.75 (95% CI, 2.55–2.95; *P* <.0001), respectively. **Figure 2** shows the significant increasing cumulative incidence of AD stratified by PPI users and nonusers with 12-month follow-up. Difference in AD developments were found for both systemic and organ-specific ADs.

**Table 2:** Risk of autoimmune disorders between PPI and non-PPI users.

Types of Autoimmune Disorders	Event	IR	IRR	Adj. HR	95% C.I. for adj. HR
Overall					
non-PPI	7592	274.67	ref.	ref.	
PPI	2926	1219.94	4.440	3.640***	3.484–3.804
Systemic					
non-PPI	4635	167.65	ref.	ref.	
PPI	1858	772.14	4.606	4.335***	4.103–4.580
Organ-specific					
non-PPI	2973	107.38	ref.	ref.	
PPI	1075	445.97	4.148	2.750***	2.558–2.957

PPI, proton pump inhibitors. Adj HR, adjusted hazard ratio was adjusted by gender, age, comorbidities. IR, incidence rate was incidences of per 100,000 person-year. CI, confidence intervals. ***P-Value < 0.001.

### Dose-Response and Adjusted HRs of ADs in PPI and Non-PPI Users

No significant relationship was observed between the PPI dose and AD risk in the PPI users. **Table 3** shows that the aHR for overall AD developments initially increased and then decreased as PPI cDDDs increased and the largest aHR was in the group of 22–42 DDDs. A similar trend of AD development was found when the analyses were restricted to systemic or organ-specific ADs. The highest aHR for systemic AD development was found in the group with 43–98 DDDs; the highest aHR for single-organ AD development was found in the group with 22–42 DDDs. However, in general, significantly higher aHRs for development of ADs were seen in the different cDDD groups of PPI users than nonusers.

**Table 3:** The dose response between PPI and autoimmune diseases.

Types of Autoimmune Disorders	Event	IR	IRR	Adj. HR	95% C.I. for adj. HR
Overall					
0	7592	274.67	ref.	ref.	
1–21	768	1071.64	3.901	3.317***	3.078–3.573
22–42	774	1353.25	4.925	4.284***	3.977–4.614
43–98	894	1442.01	5.249	4.219***	3.932–4.527
≥99	490	999.32	3.637	2.690***	2.451–2.952
Systemic					
0	4635	167.65	ref.	ref.	
1–21	483	672.06	4.009	3.789***	3.449–4.163
22–42	484	843.00	5.028	4.775***	4.346–5.245
43–98	579	930.66	5.552	5.193***	4.758–5.668
≥99	312	635.17	3.789	3.522***	3.135–3.956
Organ-specific					
0	2973	107.38	ref.	ref.	
1–21	289	401.41	3.734	2.688***	2.380–3.035
22–42	292	507.33	4.721	3.549***	3.144–4.005
43–98	316	506.60	4.712	2.999***	2.665–3.375
≥99	178	361.96	3.366	1.817***	1.558–2.120

PPI, proton pump inhibitors. Adj. HR, adjusted hazard ratio was adjusted by gender, age, comorbidities. IR, incidence rate was incidences of per 100,000 person-year. CI, confidence intervals. ***P-Value < 0.001.

### Adjusted HRs of ADs and Subgroup ADs in PPI and Non-PPI Users

In **Table 4**, as compared with the non-PPI group, the adjusted hazard ratio (aHR) were higher for incident organ-specific ADs such as Diabetes mellitus type 1 (aHR=2.65, 95%CI 2.40 to 2.93), Graves disease (aHR=3.28), Hashmoto thyroiditis (aHR=3.61), autoimmune hemolytic anemia (aHR=8.88), immune thrombocytopenic purpura (aHR=5.05) Henoch-Schonlein pupura (aHR=4.83) and Myasthenia gravis (aHR=8.73). Furthermore, the adjusted hazard ratio (aHR) were also higher for incident systemic ADs such as ankylosing spondylitis (aHR=3.67), rheumatoid arthritis (aHR=3.96), primary Sjogren syndrome(aHR=7.81), systemic lupus erythemtoasus (aHR=7.03). systemic vasculitis (aHR=5.10), psoriasis (aHR=2.57), systemic scleroderma (aHR=15.85) and inflammatory myopathy(aHR=37.40). In summary, the highest aHRs for ADs development in patients with PPI compared with patients without PPI, were inflammatory myopathy (aHR=37.40).and autoimmune hemolytic anemia(aHR=8.88).

**Table 4:** Risk of organ-specific and systemic autoimmune diseases between PPI and non-PPI users.

	Event	Incidence Rate	IRR	ADJ. HR	95% CI.
Systemic autoimmune disease					
Ankylosing spondylitis					
non-PPI	3211	116.13	1.000	1.000	
PPI	1051	435.87	3.753	3.670***	3.418–3.941
Rheumatoid arthritis					
non-PPI	809	29.25	1.000	1.000	
PPI	324	134.16	4.587	3.968***	3.482–4.523
Sjögren syndrome					
non-PPI	307	11.09	1.000	1.000	
PPI	236	97.68	8.804	7.810***	6.569–9.285
Systemic lupus erythematosus					
non-PPI	182	6.58	1.000	1.000	
PPI	116	47.98	7.296	7.029***	5.520–8.952
Systemic vasculitis					
non-PPI	49	1.77	1.000	1.000	
PPI	27	11.16	6.305	5.099***	3.162–8.225
Psoriasis					
non-PPI	47	1.70	1.000	1.000	
PPI	12	4.96	2.922	2.568**	1.348–4.891
Systemic Sclerosis					
non-PPI	33	1.19	1.000	1.000	
PPI	53	21.92	18.380	15.851***	10.190–24.660
Inflammatory myopathy					
non-PPI	15	0.54	1.000	1.000	
PPI	52	21.50	39.674	37.397***	20.920–66.870
Organ-specific autoimmune disease					
Graves’ disease					
non-PPI	152	5.49	1.000	1.000	
PPI	44	18.19	3.313	3.280***	2.335–4.608
Hashimoto’s thyroiditis					
non-PPI	1160	41.95	1.000	1.000	
PPI	382	158.25	3.773	3.606***	3.206–4.057
Autoimmune hemolytic anemia					
non-PPI	67	2.42	1.000	1.000	
PPI	82	33.91	14.008	8.877***	6.293–12.520
Immune thrombocytopenic purpura					
non-PPI	137	4.95	1.000	1.000	
PPI	71	29.36	5.932	5.048***	3.745–6.803
Henoch-Schonlein purpura					
non-PPI	98	3.54	1.000	1.000	
PPI	47	19.44	5.489	4.829***	3.383–6.886
Myasthenia gravis					
non-PPI	56	2.02	1.000	1.000	
PPI	43	17.78	8.788	8.733***	5.836–13.070

PPI, proton pump inhibitors. Adj. HR, adjusted hazard ratio was adjusted by gender, age, comorbidities. IR, incidence rate was incidences of per 100,000 person-year. CI, confidence intervals. **0.001 ≤ P-Value < 0.01, ***P-Value < 0.001.

### Cumulative Incidence of ADs and Subgroup ADs in PPI and Non-PPI Users

A Kaplan–Meier analysis revealed the cumulative incidence of ADs and subgroup ADs development in those PPI and non-PPI users (**Figure 2**). The cumulative incidence of overall, systemic and organ-specific ADs in PPI users was significantly higher than in the non-PPPI users (log-rank p value <0.0001, **Figures 2A–C**).

## Discussion

According to our review of the relevant literature, this study is the first nationwide population-based work to evaluate the relationship between PPIs and ADs. In the present study, the incidence rate of overall ADs was 3.9 times higher in the PPI users than in non-PPI users, with an adjusted HR of 3.32 after adjustment for age, sex and comorbidity. Furthermore, we found that PPI users also had an increased risk of organ-specific and systemic ADs, respectively.

Our study indicated an association between PPI and the risk of ADs, including systemic and single-organ ADs. The possible mechanism of the association of the prescription of PPIs and the development of ADs is hypothesized to be an alteration of the gut microbiome by PPIs, which leads to AD development. The effect of the host microbiota on the immune system has been identified (15). The administration of PPIs induced gut dysbiosis or disruption of the microbial balance. The alteration of the gut microbiota early in the course of PPI use was discovered in previous study (13). Alteration of the microbiome causes changes of the host immune system and then induces the development of AD. Our study provides clinical evidence to connect PPI use, the gut microbiota, and ADs.

The prescription of antibiotics altered the microbiota of the host, giving rise to the development of ADs, such as rheumatoid arthritis, multiple sclerosis, and inflammatory bowel disease (22). Bacterial or viral infection is also a major trigger of autoimmunity (23). We excluded patients receiving these medications, such as antibiotics, antivirals, and anti-tuberculosis agents, to eliminate the confounding factors. In addition, the *H. pylori* infection altering the gut microbiota was reported (24). The *H. pylori* induces a host-specific T-cell response, leading to autoimmunity *via* molecular mimicry (25). We also excluded patients with *H. pylori* infection, identified as patients receiving *H. pylori* eradication treatment.

Our study data was derived from a real-world database, the Taiwan NHIRD, and investigated that PPIs can induce an elevated risk of ADs. However, the development of ADs is affected by many factors. The microbiota play a role in the pathogenesis of ADs (15, 26). However, the evidence for dysbiosis leading to ADs have been provided by basic studies and animal models. Some evidence has been provided by human study (26). Our study hypothesized that the administration of PPIs influences the microbiome and results in imbalance of the immune system. Our results provide confirmatory evidence in real-world settings. Our study is to ascertain the association between PPIs and ADs, even though the relationships between PPIs and gastrointestinal microbiota and between gastrointestinal microbiota and host immune system have been identified. A significant increasing aHR of AD development was found in PPI users in the follow-up period, but we were unable to find a dose–response association. The aHR for the risk of ADs did not significantly increase when the PPI dose elevated. The highest aHR of overall AD development was observed in the 43–98 DDDs in patients with PPIs, rather than in the more than 99 DDDs. Even though no dose–response association was detected, the aHRs were significantly increased in different cDDD groups of PPI prescriptions compared with nonusers. These results support that PPIs induce development of ADs.

Several previous studies described the risk of several ADs is increased by PPIs. Polymyositis was found in patients treated with PPIs in the World Health Organization adverse drug reactions database (VigiBase) (27). Clarks and colleagues (28) described that 69 of 292 myopathy events recovered post PPIs withdrawn in a New Zealand study. Twenty-seven of 292 cases of myopathy were identified as polymyositis or myositis. Use of PPIs induced 24 events of subacute cutaneous lupus erythematosus on an incubation time of 8 months in Danish retrospective study (27). Aggarwal and colleagues (29) also reported that subacute cutaneous lupus erythematosus was induced by PPIs, with an elevated proportional reporting ratio of 36.64 in an analysis of U.S. FDA Adverse Event Reporting System. In a recent study based on Taiwan NHIRD, Chen and colleagues (30) reported that an incidence rate of systemic immune diseases in patients receiving PPI was 1.29 per 1000 person-years and the aHR of ADs was 1.5 in patients using PPI compared to nonusers. However, this study was limited to patients with gastric diseases and analyzed fewer systemic ADs than our study. They only analyzed the ADs of rheumatoid arthritis, systemic lupus erythematosus, Sjögren syndrome, psoriasis, polymyositis, and scleroderma. The AD events analyzed in our research are not only systemic ADs, but also single-organ ADs. In addition, the database analyzed in our research, which included almost all adults in Taiwan, is larger than the Chen’s study. We report a higher aHR of Sjögren syndrome in patients receiving PPI than the Chen’s study, which is 8.54 and 1.82 in our and Chen’s study, respectively. The aHR of rheumatoid arthritis in our study is 4.54, which is also higher than 2.19 in the Chen’s study. Our study provides data on the more complete association between different ADs, including systemic and single-organ, and the prescription of PPIs. Our result provides firmer evidence that PPIs can induce AD development.

The etiology of ADs remains unknown. We have described new potential medical factors leading to induction of ADs. Using PPIs will increase the risk of ADs. In previous studies, the prescription of PPIs had a low risk for users. Inappropriate prescription of PPIs was declared previously (31). Although they are relatively safe drugs, PPIs should be used more carefully (32). Gradually increasing adverse effects of PPIs have been identified. In our retrospective cohort study, the risk of ADs was increased by prescribing PPIs. The use of PPIs should be considered carefully for the correct indications and in select patients to prevent unnecessary overuse. Our study also raises important concerns about further therapeutic options for preventing ADs.

The limitation of our study was that we did not use a matched sample. We used nonmatching to compare data to explain a high risk of ADs in patients with PPI use in real-world settings. Difference in baseline characteristics exists between PPI users and nonusers. However, the high aHR would direct the real elevated risk for developing ADs in patients receiving PPIs. Another limitation of our study was that we cannot exclude all the possible factors affecting the gut microbiota. Although we excluded patients receiving antibiotics, antiviral agents, and antituberculosis agents, the microbiota will also be affected by a variety of host and environmental factors. We also excluded patients with major organ dysfunction diseases, including diseases of the liver and kidney. We tried to eliminate possible confounding factors by our established exclusion criteria. In addition, we could not confirm the exact time when a prescription of PPIs induced alteration of the gut microbiome. Furthermore, we could not predict the time when using PPIs induces the development of ADs. Therefore, the development of ADs was recorded as the event immediately after the prescription of PPIs in our study.

In conclusion, in the findings of our study, PPIs was associated with higher risk of the development of ADs. Therefore, it is recommended that awareness of increased risk of ADs in patients with PPI treatment is very important for clinician. Furthermore, the mechanism of PPIs inducing ADs needs further research to elucidate.

## Data Availability Statement

The original contributions presented in the study are included in the article/**Supplementary Material**. Further inquiries can be directed to the corresponding author.

## Ethics Statement

The present research was approved by the Institutional Review Board of Taipei Medical University (TMU JIRB-N201908055). Written informed consent for participation was not required for this study in accordance with the national legislation and the institutional requirements.

## Author Contributions

Conception and design of the study: S-HL, J-HC, and C-CC. Analysis and interpretation of data: Y-CL and J-HC. Drafting of article or revising it critically for important intellectual content: S-HL, Y-SC, T-ML, L-FH, T-YH, H-CH, P-IK, W-SC, Y-CL, J-HC, and C-CC. All authors contributed to the article and approved the submitted version.

## Funding

This study was supported by research grants from Taipei Medical University(TMU106-AE1-B02).

## Conflict of Interest

The authors declare that the research was conducted in the absence of any commercial or financial relationships that could be construed as a potential conflict of interest.

## Publisher’s Note

All claims expressed in this article are solely those of the authors and do not necessarily represent those of their affiliated organizations, or those of the publisher, the editors and the reviewers. Any product that may be evaluated in this article, or claim that may be made by its manufacturer, is not guaranteed or endorsed by the publisher.
