# Supplementary material for: Proton Pump Inhibitors Increase the Risk of Autoimmune Diseases: A Nationwide Cohort Study
Source: Front Immunol. 2021 Sep 30;12:736036. doi: 10.3389/fimmu.2021.736036 (PMC8514990; doi:10.3389/fimmu.2021.736036)
Supplement: Supplementary file 1 [file Table_1.docx]

Supplementary Table S1. The ICD-9-CM codes of autoimmune disease

| Systemic Autoimmune Diseases | |
| --- | --- |
| Disease | ICD 9 CM code |
| Ankylosing Spondylitis | 720 |
| Rheumatoid arthritis | 714.0, 714.30–714.33 |
| Sjögren syndrome | 710.2 |
| Systemic lupus erythematosus | 710 |
| Systemic vasculitis (Polyarteritis nodosa, Temporal arteritis, and Takayasu arteritis) | 446.0, 446.5, 446.7 |
| Psoriasis | 696 |
| Systemic sclerosis | 710.1 |
| Inflammatory myopathy (Dermatomyositis and Polymyositis) | 710.3, 710.4 |
| Single-organ Autoimmune Diseases | |
| Disease | ICD 9 CM code |
| Addison’s disease | 255 |
| Diabetes mellitus type 1 | 250.01 |
| Graves’ disease | 242 |
| Hashimoto’s thyroiditis | 245.2 |
| Autoimmune hemolytic anemia | 283 |
| Immune thrombocytopenic purpura | 287.31 |
| Henoch-Schonlein purpura | 287 |
| Autoimmune hepatitis (Lupoid hepatitis) | 571.42 |
| Myasthenia gravis | 358 |
| Inflammatory bowel disease (Regional enteritis-unspecified, Ulcerative colitis, and Behçet’s disease) | 555.9, 136.1 |
